# Duckweeds: their utilization, metabolites and cultivation

**DOI:** 10.1186/s13765-021-00644-z

**Published:** 2021-10-19

**Authors:** GahYoung Baek, Maham Saeed, Hyung-Kyoon Choi

**Affiliations:** College of Pharmacy, Chung-Ang University, Seoul, 06974 Republic of Korea

**Keywords:** Duckweed, Utilization, Metabolite, Cultivation method

## Abstract

Duckweeds are floating plants of the family *Lemnaceae*, comprising 5 genera and 36 species. They typically live in ponds or lakes and are found worldwide, except the polar regions. There are two duckweed subfamilies*—*namely *Lemnoidea* and *Wolffioideae*, with 15 and 21 species, respectively. Additionally, they have characteristic reproduction methods. Several metabolites have also been reported in various duckweeds. Duckweeds have a wide range of adaptive capabilities and are particularly suitable for experiments requiring high productivity because of their speedy growth and reproduction rates. Duckweeds have been studied for their use as food/feed resources and pharmaceuticals, as well as for phytoremediation and industrial applications. Because there are numerous duckweed species, culture conditions should be optimized for industrial applications. Here, we review and summarize studies on duckweed species and their utilization, metabolites, and cultivation methods to support the extended application of duckweeds in future.

## Introduction

Duckweeds are among the smallest free-floating aquatic plants worldwide. They have a simple morphology, comprising a few fronds; furthermore, they rarely flower [1, 2]. Duckweeds replicate and proliferate rapidly. The reproduction period is only 1.2 days per generation [3]. Additionally, they are highly adaptable and occur in diverse aquatic environments [4]. Similar to other aquatic plants, duckweed species generally inhabit the natural environment, such as ponds and lakes, and grow best especially in tropical and temperate regions [5, 6]. They can also grow well in local and industrial wastewater [7, 8]. Because of these features, duckweeds are suitable for various experimental and practical applications that require fast and high productivity.

Duckweeds have been utilized for food, pharmaceutical, phytoremediation, and other industrial applications [9–12]. Climate crisis has become a serious problem that threatens the food and feed supply of the increasing population of the world. It is known that duckweeds contain essential nutrients such as proteins, carbohydrates, and fats. Additionally, they contain a variety of secondary metabolites that are beneficial to humans. Therefore, consideration of cultivation methods of duckweeds is vital to their enhanced utilization in various industrial applications. There have been several reports regarding utilization, metabolites, and cultivation of duckweeds; these should be reviewed and summarized as a fundamental information for enhanced duckweed application. The objective of this review was to summarize the diverse utilization of duckweeds and their metabolites and cultivation methods. This review will be useful for further industrial applications of duckweeds.

## Duckweeds and their utilization

### Duckweed species

Duckweeds belong to the family *Lemnaceae*, comprising 5 genera and 36 species under two subfamilies *Lemnoidea* (15 species) and *Wolffioideae* (21 species) [13, 14]. Duckweed species self-replicate genetically identical clones in a vegetative manner, wherein daughter fronds sprout from the mother fronds [4, 15]. However, the two subfamilies differ in how they multiply. *Lemnoidea* (genera *Landoltia*, *Lemna*, and *Spirodela*) has two meristematic regions in the mother frond. In contrast, *Wolffioideae* (genera *Wolffia* and *Wolffiella*) has a single meristematic pouch in the mother frond [16, 17]. *Landoltia*, formerly *Spirodela punctata*, is a duckweed with two attached fronds [18, 19]. *Lemna* has one root and two to four fronds, and its length has been reported as up to 6.0 mm [18, 20]. *Spirodela*, which has the smallest genome among the duckweed family, has five large fronds from 4 to 12 mm in length [21, 22]. *Wolffia* is the most derived and smallest genus, which seldom exceeds 0.5 to 1.2 mm in length and 0.4 to 1.0 mm in width [17]. *Wolffiella* has a low flowering frequency and ranges from 1.0 to 5.0 mm in frond length [16, 23]. We found that duckweed species have different morphological characteristics.

The single species of *Landoltia—Landoltia punctata—*was formerly *Spirodela punctata* [18]. The 12 species of *Lemna* include *Lemna aequinoctialis*, *Lemna disperma*, *Lemna gibba*, *Lemna japonica*, *Lemna minor*, *Lemna minuta*, *Lemna obscura*, *Lemna perpusilla*, *Lemna tenera*, *Lemna trisulca*, *Lemna turionifera*, and *Lemna valdiviana*. There are two *Spirodela* species (*Spirodela intermedia* and *Spirodela polyrrhiza*) [1]. *Wolffia* includes 11 species: *Wolffia angusta*, *Wolffia arrhiza*, *Wolffia australiana*, *Wolffia borealis*, *Wolffia brasiliensis*, *Wolffia columbiana*, *Wolffia cylindracea*, *Wolffia elongata*, *Wolffia globosa*, *Wolffia microscopica*, and *Wolffia neglecta* [24]. The 10 species of *Wolffiella* include *Wolffiella caudata*, *Wolffiella denticulata*, *Wolffiella gladiata*, *Wolffiella hyalina*, *Wolffiella lingulata*, *Wolffiella neotropica*, *Wolffiella oblonga*, *Wolffiella repanda*, *Wolffiella rotunda*, and *Wolffiella welwitschii* [1]. Hereafter, *Lemna*, *Spirodela*, and *Wolffia* genera are abbreviated as *L.*, *S.*, and *W.,* respectively.

### Food and feed resources

It is known that most duckweeds proliferate rapidly, and the harvested yield per area is higher than the average of the major crop yields [4]. The protein production of duckweeds per harvested area was higher than that of soybean, rice, and corn; thus, it could solve the problem of farmland shortage to produce food or animal feed [25–28]. Duckweeds contain starch, fatty acid, protein, and other secondary metabolites used in food and feed industries [7, 9, 29–32]. Compared with red meat, plant-based foods have less of an association with cardiometabolic risks and diabetes [33, 34]. Additionally, duckweeds have been accepted as food resources without public aversion [35]. Because of their high yield, economic advantage, nutrient composition, and positive perception by people, duckweeds have been utilized as the plant-derived food and feed resources.

Table 1 lists duckweed species used as food resources. *Wolffia* species have long been consumed as protein sources by humans in Asia [9, 29, 36]. Currently, duckweeds are mainly consumed as amino acid supplements [29]. Parabel, Ltd. has a product line of duckweed plant powders as an alternative to high protein foods [37, 38]. Duckweeds are also expected to occur in the European food market [39]. Consumption of plant protein, instead of animal protein, is expected to reduce energy use and greenhouse gases [40]. Duckweeds are good candidates for nutritious and safe meat protein substitutes for humans. Consumption of *W. globosa* as a meat substitute reduces the risk of iron deficiency while maintaining iron homeostasis and folic acid concentration [41]. Clinical nutrition studies have demonstrated that the essential amino acids and vitamin B_12_ contents of duckweeds are comparable to peas and cheese [29]. Iron and zinc in duckweeds are sufficient for the recommended allowance, similar to the sodium/potassium ratio and fiber content [42]. The amino acid composition of *Wolffia* sp. and *Wolffiella* sp. meets the World Health Organization (WHO) recommendations [9, 43]. The ratio of omega-3 to omega-6 fatty acids in *Landoltia punctata*, *L*. *gibba*, *L*. *minor*, *S*. *polyrrhiza*, *W*. *microscopica*, and *Wolffiella hyalina* makes them suitable for food and feed [9]. However, it should be noted that consumption of duckweed species high in oxalic acid could cause kidney stones [44]. Moreover, intake of duckweed with substance adsorption abilities could lead to heavy metal intake.

**Table 1:** List of duckweeds used as food and feed resources

Usages	Species	References
Food		
Amino acid supplement	*W. globosa*	[29]
Iron source	*W. globosa*	[41]
Protein powder	*Lemna* spp.	[37]
Tradition food	*W. arrhiza* and *W. globosa*	[9, 29, 36]
Vitamin B_12_ supplement	*W. globosa*	[29]
Feed		
Cattle	*Lemna* spp., *Spirodela* spp., and *Wolffia* spp.	[48]
Chicken	*Landoltia punctata* and *L. gibba*	[7, 15, 26, 51]
Fish	*L. gibba, L. minor, L. perpusilla*, and *S. polyrrhiza*	[27, 46, 47, 52]
Sheep	*Landoltia punctata*	[49]
Recycled feed supplement	*L. minuta*	[45]
Waterfowl	*L. minor*	[50]

*L*. Lemna, *S*. Spirodela, *W*. Wolffia

Duckweed species have been used as livestock feed for hundreds of years and have been shown to be nutritious [15, 28, 31]. Duckweed feed can supply animals with phosphate and nitrogen [39]. *L. minuta* can be recycled as a feed supplement by adsorbing micronutrients such as selenium and zinc, which are essential for animals [45]. *W. arrhiza*, used as animal feed, yields protein content comparable to that by soybeans [17]. As listed in Table 1, duckweeds are used as feed for various livestock, including cattle, chicken, fish, sheep, and waterfowl [7, 15, 26, 27, 46–51]. *L. minor* and *S. polyrrhiza* are economically viable alternatives to fish and soybean meal feed for fish and waterfowl [27, 46, 50, 52]. In cows, there is no abnormality in the digestion of dry matter and crude protein of duckweed; thus, it could be used as an alternative feed for soybean meal [48]. Soybeans are most commonly used as feed, but the expansion of cultivation because of increased demand could emit significant greenhouse gases from land use change [53]. Duckweeds are considered as novel ingredients to replace soybeans, thus reducing the burden of greenhouse gas emissions and alleviating the negative aspects of feed production [51]. The protein content of duckweeds grown in organic manure is very high, and utilization of duckweed as feed has been suggested as a solution to environmental issues related to manure purification and feed production [54]. Duckweeds are protein sources that could replace soybean meal and are expected to be used as substitutes to reduce environmental pollution created by expanding soybean cultivation.

### Pharmaceutical resources

As represented in Table 2, duckweeds have been suggested as pharmaceutical resources. Previous studies have been reported that duckweed species such as *L. minor*, *L. trisulca*, and *S. polyrrhiza* have been widely utilized as folk medicine in China, Korea and a few European nations [12, 44, 55]. Duckweeds are medicinal herbs that do not have severe side effect [44]. Recent research has revealed the various pharmacological effects of duckweeds. *L. minor* has antibacterial activity against gram-negative bacilli (*Pseudomonas fluorescens, Shigella flexneri, Escherichia coli*, and *Salmonella typhi*) and gram-positive bacteria (*Bacillus subtilis*), and could be an alternative to antibacterial agents for the treatment of various diseases [44, 56, 57]. *S. polyrrhiza* also showed antimicrobial activities against seven gram-negative bacilli, one gram-positive bacterium, and two fungal pathogens [58]. Flavonoids in duckweeds could contribute metabolites for the antioxidant activity [59]. Apigenin and vitexin in *Landoltia punctata* have been suggested as constituents for treating non-small lung cancer [59]. *L. minor* has been shown to have immunomodulatory activity [57]. In particular, flavonoids in *L. minor* have been reported to have immunosuppressive effects by reducing free hemoglobin content and antibody production in human whole blood samples infected with ovalbumin antigen [60]. Flavonoids in *S. polyrrhiza* have been demonstrated to exert anti-adipogenic effect by reducing triglyceride content [61]. An increase in fecal butyric acid has been reported as a result of duckweed consumption, which may be associated with improved colon health in humans and is an important energy source for colon cells [62]. Although further research and clinical trials are required for practical use, duckweeds have great potential to be utilized for pharmaceutical purposes because of their diverse pharmacological effects.

**Table 2:** List of duckweeds used as pharmaceutical resources

Pharmaceutical activities	Species	References
Antibacterial activity	*L. minor* and *S. polyrrhiza*	[44, 56–58]
Anticancer activity	*Landoltia punctata*	[59]
Anti-adipogenic effect	*S. polyrrhiza*	[61]
Antifungal activity	*L. aequinoctialis* and *S. polyrrhiza*	[58, 69]
Antigen expression for vaccines after nuclear transformation	*L*. *minor*	[66]
Antioxidant activity	*Landoltia punctata*, *L. gibba*, *L. minor*, *S. polyrrhiza*, *W. borealis*, and *Wolffiella caudata*	[44, 59]
Colonic health improvement	*Landoltia punctata*	[62]
Cytotoxic activity	*L. minor*	[44]
Folk medicine (antiscorbutic, asthma, colds, diabetes, diuretic, febrifuge, general tonic, hives, measles, edema, rhinitis, soporific, and vitiligo)	*L. minor*	[12, 44]
Folk medicine (choleretic and phytoncidic activities)	*L. trisulca*	[12]
Folk medicine (erysipelas and leprosy)	*S. polyrrhiza*	[12]
Immunomodulatory activity	*L. minor*	[57, 60]
Monoclonal antibody production as a transgenic plant by LEX System	*L. minor*	[63, 64]
Monoclonal antibody production for non-Hodgkin’s lymphoma	*L. minor*	[67, 68]
Recombinant human granulocyte colony-stimulating factor production	*W. arrhiza*	[65]

*L*. Lemna, *S*. Spirodela, *W*. *Wolffia*, *LEX System* Lemna Expression System

Another pharmacological potential of duckweeds is their use as a platform for human therapeutic proteins production. Recombinant therapeutic proteins are produced by Lemna Expression System (LEX System) [63, 64]. Additionally, nuclear-transformed *W. arrhiza* expresses human granulocyte colony-stimulating factor [65]. Nuclear-transformed *L. minor*-based expression studies for avian influenza vaccine development have shown the potential of transgenic duckweed to provide good-quality antigens for vaccine development [66]. Synthon/Biolex Therapeutics produces large quantities of antibodies for non-Hodgkin's lymphoma using duckweed species [67, 68]. Duckweed-based antibody production could be considerably inexpensive and could provide easy to scale up platforms for diverse antibody-based therapies to treat and prevent diseases.

### Phytoremediation resources

Duckweeds, tolerant to extreme conditions, are known as effective remediation resources for pollutants in wastewater. They purify sewage through the powerful accumulation of chemicals by adsorption or uptake [25, 70, 71].

As listed in Table 3, duckweeds have been reported to purify inorganic, organic, and pharmaceutical materials. Ammonium elimination is necessary for the purification of wastewater because ammonium increases eutrophication in ponds and forms nitrates in groundwater [72]. *Landoltia punctata* absorbs ammonium from water and stores ammonium ions as a useful nitrogen source [19, 73]. Excessive concentration of boron, a by-product of industrial production, is detrimental to the ecosystem. *L. gibba* has also been reported to remove boron from water [11, 74]. Boron is adsorbed to apiogalacturonans in the cell wall of duckweeds [75]. *L. minor* captures iron, *L. gibba* captures sulfate, and *S. polyrrhiza* captures fluoride [76–78].

**Table 3:** List of duckweeds used as phytoremediation resources

Phytoremediation targets	Species	References
Inorganic		
Ammonium	*Landoltia punctata*	[19]
Arsenic	*L. gibba*, *L. valdiviana, S. intermedia*, and *W. arrhiza*	[83, 87–89]
Boron	*L. gibba*	[11, 74]
Cadmium	*L. minor* and *L. trisulca*	[90–92]
Chromium	*S. polyrrhiza*	[93]
Cobalt	*Landoltia punctata*	[94]
Copper	*L. aequinoctialis*, *L. gibba*, *L. minor*, and *L. trisulca*	[84, 85, 91, 92]
Fluoride	*S. polyrrhiza*	[78]
Iron	*L. minor*	[77]
Lead	*L. perpusilla*	[86]
Nickel	*Landoltia punctata* and *L. minor*	[90, 94]
Nitrogen	*Landoltia punctata*, *L. gibba, L. minuta*, and *S. polyrrhiza*	[19, 70, 76, 79–81]
Phosphorous	*Landoltia punctata*, *L. gibba, L. minuta*, *L. turionifera*, *S. polyrrhiza*, and *W. borealis*	[70, 71, 76, 79–81]
Selenium	*L. minuta*	[45]
Sulfate	*L. gibba*	[76]
Zinc	*L. minor* and *L. minuta*	[45, 90]
Organic		
Dairy industry processing wastewater	*L. minor*	[100]
Hydrocarbons in crude oil-contaminated wetlands	*L. aequinoctialis*	[101]
Synthetic dyes	*L. minor*	[102]
Wastewater	*Landoltia punctate* and *W. arrhiza*	[7, 17]
Pharmaceutical		
Acetaminophen	*L. turionifera* and *W. borealis*	[71]
Antimicrobials	*L. minor*	[99]
Benzotriazoles	*L. minor*	[95]
Fluoxetine	*L. turionifera* and *W. borealis*	[71]
Ibuprofen	*L. gibba*	[97]
Imidacloprid	*L. turionifera*	[96]
Progesterone	*L. turionifera* and *W. borealis*	[71]
Sulfamethoxazole	*L. turionifera* and *W. borealis*	[71]
Tetracycline antibiotics	*L. gibba*	[98]

*L*. Lemna, *S*. Spirodela, *W*. Wolffia

Nitrogen and phosphorous emissions from manure in livestock systems contribute to eutrophication of ecosystems; thus, their recovery and reuse are significant and essential tasks [79]. For example, *Landoltia punctata, L. gibba, L. minuta, L. turionifera, S. polyrrhiza, and W. borealis* purify wastewater or swine lagoons by removing nitrogen and phosphorous [19, 70, 71, 76, 79–81]. The adsorption capacity of nitrogen and phosphorus is especially valuable because duckweeds can be reused as fertilizers that release nitrogen and phosphorus in the soil [82].

Duckweeds are resistant to several heavy metals and can be used for bioremediation in local and industrial wastewater by accumulating heavy metals, including arsenic, cadmium, chromium, cobalt, copper, lead, nickel, selenium, and zinc [45, 83–94]. They have an enzymatic antioxidant mechanism to control oxidative stress caused by heavy metals and reduce damage [83]. Duckweeds relieve the stress of heavy metals that affect nutrient absorption by activating antioxidative mechanisms [85]. The chelating action of duckweeds can also alleviate the stress caused by heavy metals, such as chromium (IV) [85, 93].

The release of diverse pharmaceuticals into environment severely affects various plants and animals, and their removal through phytoremediation is important. *L. turionifera* and *W. borealis* could remove pharmaceuticals, such as acetaminophen, fluoxetine, progesterone, and sulfamethoxazole [71]. *L. minor* can remove benzotriazoles used in anti-corrosion products, coolants, and dishwashing liquids [95]. *L. turionifera* can eliminate pesticides, such as imidacloprid insecticide, from contaminated water [96]. *L. gibba* was used for the remediation of fresh water contaminated with nonsteroidal anti-inflammatory drug ibuprofen [97]. Applications of *L. gibba* for removing tetracycline and *L. minor* for removal antimicrobials have also been reported [98, 99].

Industrial wastewater from modern factories and sewage from large animal farms, hospitals, and homes are known to cause problems in aquatic environments. Duckweeds could be the best aquatic purification plant. Phytoremediation using duckweeds is a cost-effective and environmentally friendly strategy to prevent environmental pollution and preserve aquatic and terrestrial ecosystems. However, the purification capacity of duckweeds and disposal of contaminated duckweeds should be thoroughly considered.

### Biofuels, space exploration, and bioplastics

In recent years, interest in the developing alternative energy sources has increased owing to environmental problems and climate changes. Biofuels are renewable energy alternatives to fossil fuels and include bioalcohols, biodiesel, and biogas. Duckweeds have been used for bioethanol production as a renewable energy resource [25]. With the increasing need for biomass, research has focused on duckweeds and their starch content. Under proper conditions, the doubling time of duckweed biomass ranges from 1.34 to 4.54 days [4]. Starch could be used for bioethanol production through a considerably simple conversion process [25]. Duckweed biomass production is economical because, unlike corn, duckweeds do not require mechanical grinding; additionally, the by-product of ethanol fermentation has a high protein content that could be reused as livestock feed [25].

As listed in Table 4, duckweeds have been used as a resource for biomass and biofuel production. Duckweeds with a high starch content, high biomass production, and low lignin content could be promising sources of bioethanol production [103]. Duckweeds can be enzymatically converted to bioethanol by fermentation without thermophysical pretreatment [104]. *L. aequinoctialis* and *S. polyrrhiza* have been selected to increase bioethanol yield as they have a high starch content and biomass production capacity [105, 106]. *S. polyrrhiza* could be utilized as a substitute for corn starch to make the bioethanol industry sustainable because its ethanol yield (6.42 × 10^3^ l ha^−1^) has been found to be higher than that of corn (4.31 × 10^3^ l ha^−1^) [106]. Cytokinin treatment has been suggested as a good option to increase the growth rate of and starch accumulation in *S. polyrrhiza* [107].

**Table 4:** List of duckweeds used in biofuels and bioplastics applications

Usages	Species	References
Biobutanol	*Landoltia punctata*	[103]
Biodegradable plastics for various industrial products	*Lemna* sp.	[115]
Bioethanol	*L. aequinoctialis*, *L. minor*, and *S. polyrrhiza*	[104–106]
Biofuels	*L. gibba*, *S. polyrrhiza*, and *W. australiana*	[109]
Biogas	*L. minor*	[108]
Biomass production as a bioregenerative system for space exploration	*L. aequinoctialis*, *L. gibba*, and *W. globosa*	[111, 112]
Photosynthetic producer in space exploration	*W. arrhiza*	[113]
Plant fuel cell	*L. minuta*	[110]

*L*. Lemna, *S*. Spirodela, *W*. Wolffia

*Landoltia punctata* could be used for biobutanol production by a mutant strain of yeast [103]. *L. minor* can be pyrolyzed to produce biochar, and biochars are catalysts for biogas [108]. After extracting starch from duckweeds, the residual cell wall can be broken down into sugars and uronic acids, which can be converted into biofuel sources [109]. Additionally*, L. minuta* has been reported as an eco-friendly energy resource that converts solar energy into electricity by acting as a plant fuel cell to generate electricity [110].

*L. aequinoctialis* and *W. globosa* have a high relative growth rate even under microgravity, making them suitable for use in space exploration [111]. *L. gibba* is also suitable for agriculture and bioregeneration systems for space exploration because it has shown a high growth rate under an extreme range of lighting from low growth luminosity to a total daily photon mass similar to that received on the brightest and longest days [112]. In long-term space exploration, *W. arrhiza* could also be a photosynthetic producer [113].

Duckweeds can be utilized as resources for biodegradable plastics. Biodegradable plastics are polymers that can be degraded by living organisms and are invented as alternatives to non-degradable plastics [114]. *Lemna* species produce biodegradable plastics for various industrial products [115]. Blending duckweed biomass and polyethylene has shown good stability and matrix characteristics [115].

## Useful metabolites in duckweeds

### Proteins, carbohydrates, and fats in duckweeds

Table 5 lists the total content of proteins, carbohydrates, and fats in various duckweeds. The proportion of the total content of amino acids in duckweeds was the highest compared with that of carbohydrates and fatty acids. The total protein content of duckweeds ranges from 19.8% to 48.2% per dry weight. Relatively higher level of total amino acid (48.2%) per dry weight was observed in *W. globosa* [116]. Relatively higher level of total carbohydrate content (38%) was reported in *L. gibba* [117]. In addition, total fatty acid content was relatively higher in *L. minor* (11.4%) [30].

**Table 5:** Total contents of proteins, carbohydrates, and fats in duckweeds

Duckweed species	Proteins	Carbohydrates	Fats	References
Landoltia				
*Landoltia punctata*	20–28.7%	10–11.20% (Starch)	4–5.5%	[9, 118, 119]
Lemna				
*L. aequinoctialis*	34.18%	11.61–28.68% (Starch)	NS	[69, 105, 119]
*L. gibba*	21.5–37.9%	17.6–38.0%	4.4–9.3%	[117, 118, 120]
*L. minor*	20–38.30%	4% (Starch)	11.4%	[9, 30, 42]
Spirodela				
*S. polyrrhiza*	25.6–34.5%	11.14–29.8% (Starch)	4.5%	[46, 105, 106, 118, 119, 121]
Wolffia				
*W. arrhiza*	19.8–20.15%	43.6%	2.43–5.0%	[36, 116]
*W. columbiana*	36.5–44.7%	NS	6.6%	[116, 118]
*W. globosa*	33.3–48.2%	11.05% (Starch)	5.0–9.6%	[116, 119]
Wolffiella				
*Wolffiella hyalina*	35%	NS	7%	[9]

*L*. Lemna, *S*. Spirodela, *W*. Wolffia, *NS* not specified

The value before (starch) represents the starch content

The nine essential amino acids found in the duckweed species are histidine, isoleucine, leucine, lysine, methionine, phenylalanine, threonine, tryptophan, and valine and eleven non-essential amino acids, namely alanine, arginine, asparagine, aspartate, cysteine, glutamate, glutamine, glycine, proline, serine, and tyrosine, were also found in duckweeds (Table 6) [9, 15, 54, 116, 122]. Amino acid derivatives, such as citrulline, cystathionine, hydroxyproline, γ-aminobutyric acid (GABA), and taurine, were also found (Table 6) [54, 122]. Notably, the amino acid compositions of *W. microscopica* and *Wolffiella hyalina* are close to the content of lysine (30 mg/kg), sulfur amino acids (15 mg/kg) including cysteine and methionine, threonine (15 mg/kg), and tryptophan (4 mg/kg) required for adults per day according to WHO recommendations. Additionally, the contents of cysteine and methionine are 22% higher than the recommended allowance [43].

**Table 6:** List of amino acids, saccharides, and fatty acids in duckweeds

Compounds		References
Proteins		
Amino acids	Alanine, arginine, asparagine, aspartate, cysteine, glutamate, glutamine, glycine, histidine, isoleucine, leucine, lysine, methionine, phenylalanine, proline, serine, threonine, tryptophan, tyrosine, and valine	[9, 15, 54, 116, 122]
Amino acid derivatives	Citrulline, cystathionine, hydroxyproline, γ-aminobutyric acid (GABA), and taurine	[54, 122]
Carbohydrates		
Sugars	Apiose, arabinose, fructose, fucose, galactose, glucose, mannose, raffinose, rhamnose, sucrose, and xylose	[10, 75]
Polysaccharides	Inulin, pectin, and hemicellulose	[109, 120]
Starch	Starch	[9, 75, 119]
Fats		
Long-chain fatty acids	Behenic acid, eicosanoic acid, 6-hexadecenoic acid, 2-hydroxypalmitic acid, lignoceric acid, α-linolenic acid, γ-linolenic acid, linoleic acid, linolenic acid, myristic acid, nonadecylic acid, oleic acid, palmitelaidic acid, palmitic acid, pentadecylic acid, stearic acid, and stearidonic acid	[9, 17, 32, 119, 120, 123, 124]
Medium-chain fatty acids	Lauric acid	[120, 123]
Short-chain fatty acids	C2, C3, C4, and C5	[28]

Carbohydrates in duckweeds comprised sugars, polysaccharides, and starch. Sugars, such as apiose, arabinose, fructose, fucose, galactose, glucose, mannose, raffinose, rhamnose, sucrose, and xylose, were found in duckweeds (Table 6) [10, 75]. The compositions of duckweed cell walls were similar among species, consisting of pectin and hemicellulose [109]. Among the polysaccharides that do not make up the cell wall, inulin has been reported [120]. Starch accounts for approximately 4.0% to 29.8% of duckweed species, as listed in Table 5. High levels of starch accumulation have been observed under nutritional deficiency conditions in *S. polyrrhiza* [106]. Adequate salinity condition (150 mM NaCl) induce starch accumulation in *Landoltia punctata*, *L. aequinoctialis*, *L. gibba*, *L. minor*, and *S. intermedia* [125]. However, duckweeds produce low total protein levels under saline condition (10, 20, and 30 mmol/L NaCl concentration) [106]. Therefore, when choosing salt treatment as an induction method to increase biomass for biofuel production, circumspection regarding its correlation with protein levels is required. *L. aequinoctialis* and *S. polyrrhiza* are known as high-starch duckweed species; therefore, they can be utilized in the biofuel industry [105, 106].

As listed in Table 5, the total fatty acids content ranges from 1.05% to 1.62% and the triacylglycerol composition is 0.02% in *Landoltia punctata*, *L. aequinoctialis*, *S. polyrrhiza*, and *W. globosa* [119]. The fatty acids found in duckweeds included behenic acid, eicosanoic acid, 6-hexadecenoic acid, 2-hydroxypalmitic acid, lauric acid, lignoceric acid, α-linolenic acid, γ-linolenic acid, linoleic acid, linolenic acid, myristic acid, nonadecylic acid, oleic acid, palmitelaidic acid, palmitic acid, pentadecylic acid, stearic acid, and stearidonic acid (Table 6) [9, 17, 32, 119, 120, 123]. Three fatty acids—linoleic acid, linolenic acid, and palmitic acid—are dominant, accounting for approximately 80% of the total fatty acids [119]. The content of α-linolenic acid is also particularly high (between 11 and 25%) [9]. The ratio of omega-3 to omega-6 fatty acids in duckweeds has been reported to range from 5:3 to 4:1 [9]. Increased consumption of omega-3 fatty acids could prevent inflammatory diseases, cancer, cardiovascular diseases, and other chronic diseases, therefore, the omega-3:omega-6 ratio in duckweeds is noteworthy [126].

Short chain fatty acids (SCFAs) have also been identified in *L. minor*, the total SCFA is 16.6 mM, of which C_2_ and C_3_ account for 11.8 and 3.1 mM, respectively [28]. SCFAs can be absorbed into colon epithelial cells through diffusion or active transport; C_2_ and C_3_, in particular, are easily transported to other cells and organs [127]. SCFAs have been reported to contribute to the healthy intestinal environment, regulate the immune system, and prevent colorectal cancer [127, 128]. Despite their substantial potential as bioactive materials, profiling individual intact lipid species in duckweeds has rarely been performed. Further investigation should be conducted to reveal the profiles of such lipid species in various duckweed species to broaden application of duckweeds.

### Secondary metabolites in duckweeds

As listed in Table 7, duckweeds contain various useful secondary metabolites including phenolic compounds (flavonoids, phenylpropanoids, and tannins) and terpenoids. Various physiological properties have facilitated their utility, and they have been highlighted in the pharmaceutical, cosmetics, and food industries.

**Table 7:** Secondary metabolites in duckweeds

		Contents	Species	References
Phenolic compounds	Flavonoids	Anthocyanin	*L. gibba* and *S. intermedia*	[83, 87]
		Apigenin	*Landoltia punctata* and *S. polyrrhiza*	[59, 131]
		Apigenin 6-*C*-(2″-*O*-trans-caffeoyl-D-malate)-β-glucoside	*L. japonica*	[132]
		Apigenin 7‐*O*‐glucoside	*S. polyrrhiza*	[59, 131]
		Apigenin 8‐*C*‐glucoside (vitexin)	*Landoltia punctata*, *L. gibba*, and *S. polyrrhiza*	[59, 131]
		5-*O*-(E)-caffeoylquinic acid	*Landoltia punctata* and *S. polyrrhiza*	[59]
		3-*O*-(E)-coumaroylquinic acid	*S. polyrrhiza*	[59]
		6,8-Di-*C*-β-glucosylapigenin	*L. japonica*	[132]
		6,8-Di-*C*-β-glucosylluteolin	*L. japonica*	[132]
		Isoorientin	*L. japonica*	[132]
		Isovitexin	*L. japonica*	[132]
		Luteolin	*S. polyrrhiza*	[131]
		Luteolin-6-*C*-glucoside-8-*C*-rhamnoside	*Landoltia punctata* and *W. borealis*	[59]
		Luteolin 6-*C*-(2″-*O*-trans-caffeoyl-D-malate)-β-glucoside	*L. japonica*	[132]
		Luteolin 6-*C*-(2″-*O*-trans-coumaroyl-D-malate)-β-glucoside	*L. japonica*	[132]
		Luteolin 7‐*O*‐glucoside	*S. polyrrhiza*	[59, 131]
		Luteolin-7-*O*-β-glucoside	*L. japonica*	[132]
		Luteolin-7-*O*-glucoside-*C*-glucoside	*Landoltia punctata*, *W. borealis*, and *Wolffiella caudata*	[59]
		Luteolin 8‐*C*‐glucoside (orientin)	*S. polyrrhiza* and *W. borealis*	[59, 131]
		Luteolin-8-*C*-glucoside-6-*C*-rhamnoside	*Landoltia punctata*, *L. gibba*, *W. borealis*, and *Wolffiella caudata*	[59]
		Luteolin-8-*C*-glucoside-6-*C*-xyloside	*Landoltia punctata*, *L. gibba*, and *W. borealis*	[59]
	Hydroxycinnamic acids	Caffeic acid	*L. aequinoctialis* and *W. arrhiza*	[17, 133]
		Cinnamic acid	*S. polyrrhiza*	[134]
		m-Coumaric acid	*L. aequinoctialis*	[133]
		p-Coumaric acid	*L. aequinoctialis*, *L. minor*, and *W. arrhiza*	[17, 123, 133]
		Ferulic acid	*L. minor* and *W. arrhiza*	[17, 123]
		Isoferulic acid	*L. aequinoctialis*	[133]
		Sinapic acid	*L. aequinoctialis*	[133]
	Tannin	Tannin	*L. aequinoctialis*, *L. minor*, and *S. polyrrhiza*	[28, 58, 69]
Terpenoids	Diterpenoid	Neophytadiene	*W. arrhiza*	[17]
	Triterpenoids	Δ5-Avenasterol	*W. microscopica*	[9]
		Campesterol	*L. aequinoctialis*, *L. minor*, *S. polyrrhiza, W. arrhiza*, and *W. microscopica*	[9, 17, 124, 133, 134]
		Cycloartenol	*W. microscopica*	[9]
		24-Methylenecycloartan-3-one	*W. arrhiza*	[17]
		Saponin	*L. minor* and *S. polyrrhiza*	[28, 42, 58]
		β-Sitosterol	*L. aequinoctialis*, *S. polyrrhiza*, *W. arrhiza*, and *W. microscopica*	[9, 17, 133, 134]
		δ-Sitosterol	*L. minor*	[124]
		Stigmasterol	*L. aequinoctialis*, *L. minor*, *S. polyrrhiza*, *W. arrhiza*, and *W. microscopica*	[9, 124, 133, 134]
		Squalene	*S. polyrrhiza*	[134]
	Tetraterpenoids	α-Carotene	*L. gibba*	[120]
		β-Carotene	*L. gibba*, *L. minor*, and *W. microscopica*	[9, 30, 112, 120]
		Loliolide	*L. minor*	[124]
		Lutein	*L. gibba* and *W. microscopica*	[9, 112, 120]
		Lycopene	*L. gibba*	[120]
		Violaxanthin	*L. gibba* and *W. microscopica*	[9, 112, 120]
		Xanthophyll	*L. gibba*	[120]
		Zeaxanthin	*L. gibba* and *W. microscopica*	[9, 112]

*L*. Lemna, *S*. Spirodela, *W*. Wolffia

Phenolic compounds are bioactive compounds with diverse pharmacological activities in humans [129]. The total phenolic content ranges from 1.3% to 2.9% in *L. minor* [30]. As listed in Table 7, the phenolic compounds detected in duckweeds are flavonoids, hydroxycinnamic acids, and tannin. The flavonoids in duckweeds are apigenin, luteolin, and their derivatives (Table 7). Duckweeds are known to have a higher flavonoid content (> 2%) than most other plants (0.5% to 1.5%) [94]. In particular, *S. polyrrhiza* and *W. globosa* has a high flavonoid content (4.22% and 5.85%, respectively), which could be advantageous in producing flavonoids [130]. *Landoltia punctata* showed significant apigenin content, and the contents of luteolin and its derivatives were high in *L. gibba*, *S. polyrrhiza*, *W. borealis*, and *Wolffiella caudata* [59]. *Landoltia punctata*, *S. polyrrhiza*, *W. borealis*, and *Wolffiella caudata* contain abundant *C*-glycosylated flavonoids, which exhibit high antioxidant activity [59, 131]. Apigenin and vitexin of *Landoltia punctata* could be used as anticancer adjuvants, and flavone *C*- glycosides from *L. japonica* exhibits cytotoxic activity against various human cancer cell lines, such as HepG-2, SW-620, and A-549 [59, 132]. Anthocyanins have been detected in *L. gibba* and *S. intermedia* and exhibit antioxidant properties [83, 87]. Hydroxycinnamic acids detected in duckweeds include caffeic acid, cinnamic acid, m-coumaric acid, p-coumaric acid, ferulic acid, isofelulic acid, and sinapic acid [17, 123, 133, 134]. Hydroxycinnamic acids possess antibacterial, anticollagenase, anti-inflammatory, anti-obesity, antioxidant, anti-tyrosinase, neuroprotection, and ultraviolet protection activities that could contribute to human health [135, 136].

Terpenoids (isoprenoids) have been widely utilized in the pharmaceutical, food, cosmetic, pesticide, chemical industries [137]. Terpenoids such as carotenoids, phytosterols, and saponins, have been detected in duckweeds (Table 7). Neophytadiene, 24-methylenecycloartan-3-one, saponin, and squalene have also been reported [17, 28, 42, 58, 134]. Saponins, including 24-dehydroechinoside, echinoside A, stichoposide C, and stichoposide D, show antitumor, hypolipidemic activity, and antihypertensive effects and suppress fat accumulation [138]. *L. minor* contains a total saponin content 3.2 g/kg dried weight [28]. Phytosterols, such as Δ5-avenasterol, campesterol, cycloartenol, β,δ-sitosterol, and stigmasterol, have been reported in duckweeds [9, 17, 124, 133, 134]. Phytosterols account for approximately 20% of the wax fraction of *S. polyrrhiza* [134]. Phytosterols are cholesterol-lowering agents that reduce serum and liver cholesterol [139]. Carotenoids belonging to the tetraterpene found in duckweeds included α-carotene, β-carotene, loliolide, lutein, lycopene, violaxanthin, xanthophyll, and zeaxanthin [9, 30, 112, 120, 124].

## Cultivation

Duckweed species are distributed in various regions in the natural environment, except for deserts and polar regions [6]. The growth of duckweed species is exponential and faster than most other plants under appropriate carbon dioxide, light, pH, temperature, and nutrient supply conditions [15, 73]. Table 8 lists the culture conditions of various duckweeds. Erlenmeyer flasks, Magenta vessel, and Petri dishes have been used for most small-volume cultures (< 500 mL) [4, 75, 96, 100, 104, 107, 140–142, 161]. Small-scale cultures should be subcultured periodically, and the light–dark cycle and temperature conditions should be constant to achieve a uniform growth rate and constant nutrient contents.

**Table 8:** Culture conditions of duckweeds

Culture volume	Culture scale	Culture medium	Species	pH/light/temperature/culture period	Refs.
≤ 50 mL					
10 mL	Magenta vessel	1/2 SH	*L. turionifera*	pH 6.0/23 °C in light/21 °C in the dark	[96]
10 mL	Petri dish (60 × 10 mm)	1/2 SH	*L. turionifera*	23.8–29.7 °C in light/21.5 °C in dark/4 days	[140]
50 mL	Petri dish	1/2 Hutner	*L. minor* and *L. minuta*	6, 10, 20, 30, 40, 90,150, 250, 400, or 1000 μmol m^−2^ s^−1^/20 °C	[141
50–100 mL					
100 mL	Petri dish (100 × 15 mm)	Hoagland	*Lemna* sp., *Spirodela* sp., *Wolffia* sp., *Wolfiella* sp.	pH 5.8/40 μmol m^−2^ s^−1^/23 °C / 2 weeks	[161]
100 mL		1/2 SH	*Landoltia* spp., *Lemna* spp., *Spirodela* spp., *Wolffia* spp., and *Wolffiella* spp.	pH 6.5/20 or 500 μmol m^−2^ s^−1^/25 °C/1 week	[75]
100 mL	Erlenmeyer flask (250 ml)	Hoagland	*L. minor*	pH 5.8/120 μmol m^−2^ s^−1^/22 °C	[104]
100 mL	Erlenmeyer flask (500 ml)	SH	*L. gibba* and *S. polyrrhiza*	80 μmol m^−2^ s^−1^/24 °C	[107]
100 mL	Magenta vessel (77 × 77 × 97 mm)	Synthetic dairy wastewater or 1/2 Hutner	*L. minor*	pH 4.5–5 / 80.82 μmol m^−2^ s^−1^ / 21 °C / 1 week	[100]
100–500 mL					
180 mL	Erlenmeyer flask (300 mL)	Modified SH	*Landoltia* spp.*, Lemna* spp.*, Spirodela* spp.*, Wolffia* spp.*,* and *Wolffiella* spp.	pH 5.5/100 μmol m^−2^ s^−1^/25 ± 1 °C/1 week	[4]
250 mL	Flask (500 mL)	Hoagland	*L. minor*	pH 7.0/75 μmol m^−2^ s^−1^/28 °C/10 days	[142]
500–1000 mL					
600 mL	Glass beaker (2 L)	1/2 MS	*S. polyrrhiza*	pH 5.8/5000 l×/25 ± 1 °C	[149]
	Plastic box (750 mL) (12.5 × 12.5 × 4.2 cm)	SH or tap water	*L. aequinoctialis* and *S. polyrrhiza*	pH 5.8/110 μmol m^−2^ s^−1^/25 °C/1 week	[105]
1000 mL	Crystallizing dish (1200 mL) (150 × 75 mm)	SH	*L. gibba*	pH 5.5/100, 200, 500, or 700 μmol m^−2^ s^−1^/25 °C / 4 days	[112]
1 L <					
2.3 L	Glass vessel (2.5 L)	Influent wastewater or 1/50 Hutner	*L. minor* and *W. arrhiza*	pH 7.1 / 50 μmol m^−2^ s^−1^ / 25 ± 0.5 °C / 1 week	[150]
2.5 L	Plastic vessel (3 L)	1/2 Hoagland	*L. gibba*	pH 6.0–7.0/outdoor light (Sde Boker, Israel)/25 ± 6 °C	[11]
10 L	Polyethylene container (45 × 30 × 20 cm)	Modified Hoagland	*S. intermedia*	pH 6.5/250 μmol m^−2^ s^−1^/25 ± 2 °C	[83]
320 × 10^1^ L	Polyethylene mesocosm (2 × 4 × 0.4 m)	Natural drainage water or underground water	*L. gibba*	pH 5–9/outdoor light/25–35 °C	[73]
126 L and 100 × 10^3^ L	Cemented outdoor tank (1.2 × 0.35 × 0.3 m) or cemented pond (20 × 10 × 0.5 m)	Organic manure and inorganic fertilizer	*S. polyrrhiza*	pH 6.98–7.91 or pH 7.76–8.30/26.0 μmol m^−2^ s^−1^/9.4–26.7 °C or 30.5–33.0 °C/10 days	[54]
180 × 10^3^ L	Outdoor pond (300 m^2^ × 0.6 m)	Pig effluent lagoon or well water	*S. polyrrhiza*	pH 8.4 or 7.2/2.89 mmol m^−2^ s^−1^/20–30 °C	[106]
7620 × 10^3^ L	Artificially designed pond (518 × 12 × 0.3 m)	Ground water, surface water, and well water	*Lemna* spp.	Direct or indirect sunlight (Florida, United States)	[37, 162]

SH medium used was Schenk and Hildebrandt [144]. MS medium was Murashige and Skoog [146]

1/2 indicates half-strength of culture medium. Parentheses in the culture scale indicate the volume or size of the vessels

*L*. Lemna, *S*. Spirodela, *W*. Wolffia

It is important which culture medium to choose because incorrect selection can lead to physiological disturbances or death of the plants [143]. Schenk and Hildebrandt medium, Hutner medium, Murashige and Skoog medium, and Hoagland medium are generally used as refined media for the culture of various duckweeds. Schenk and Hildebrandt medium contains sucrose as the carbon source and KNO_3_ as a nitrogen source [144]. Regarding nitrogen sources, Hutner medium contains NH_4_NO_3_, Murashige and Skoog medium contains NH_4_NO_3_ and KNO_3_, and Hoagland medium contains Ca(NO_3_)_2_ and KNO_3_ [145–147]. The differences in nitrogen sources could affect the starch and biomass production of duckweeds [105, 148].

In mass production (> 500 mL), duckweeds are cultivated in artificial environments (bioreactors) or natural ponds, well water, dairy, and local wastewater [11, 37, 54, 73, 83, 105, 106, 112, 149, 150, 162]. Under optimal environment conditions, including wind protection, water nutrient concentration, and optimum duckweed density, duckweeds can produce biomass with a productivity of 10–30 tons/ha per year [6, 151]. In open pond systems, *S. polyrrhiza* in organic manure and inorganic fertilizer is high in protein and carbohydrate contents, respectively [54]. Duckweeds grow rapidly even in animal wastewater, producing high biomass [106]. In the natural underground water, duckweeds have a tendency to grow slowly, lengthen roots, and possess lower protein content because of insufficient nitrogen and mineral nutrients [6, 106]. Conditions of essential phytonutrients, such as ammonium, calcium, magnesium, nitrogen, and phosphorous, affect the biomass of duckweeds [73].

Duckweeds grow in a wide pH range of 3.5 to 9.0, and the optimal pH range is between 6.5 and 7.5 [6, 73, 152, 153]. The pH determines the ratio of NH_3_ to NH_4_^+^ in the culture medium. As the pH increases, NH_4_^+^ increases, thus preventing the transport of anions in the duckweed membrane and eventually reducing growth, high NH_3_ at low pH exhibits toxicity [73, 153]. The pH also has a significant effect on the conversion of duckweed biomass into biobutanol. Bacterial growth yields sufficient butanol for industrial use when culture maintained between pH 4.5 and 5.0 [103].

Light intensity and duckweed growth rate show a direct relationship, unless the light intensity is too high. The lowest growth rate was at a low intensity of 6 μmol m^-2^ s^−1^, and the growth rate increased with increasing light intensity to 1000 μmol m^-2^ s^−1^ [141]. Duckweeds accumulate antioxidants to prevent damage when exposed to excessive light. However, it has been proposed that the choice of optimal light intensity balances light efficiency with the antioxidant contents [112]. It was also reported that different light qualities affected the growth and physiological characteristics of duckweeds. Irradiation with ultra-high-frequency electromagnetic radiation has increased the growth rate and biomass, whereas infrared irradiation increased the number of fronds in *L. minor* culture [154]. However, ultraviolet rays delay the development of duckweeds and the growth of the root system, and radiofrequency radiation induced oxidative stress in the plants [154, 155].

In a natural environment, duckweeds generally grow in the range of 6 to 33 ℃; the optimum water temperature for duckweeds growth is between 19 and 30 ℃ [6, 73, 152]. In late summer in the temperate climate region of the world, duckweeds undergo a morphological change called turion because of the reduction in temperatures [6]. They sink into water bodies, storing starch as energy for the next growing season, and remain dormant. When the temperature increases in spring, they germinate because of light [156, 157]. Turion-type duckweeds could be suggested as useful biofuel feedstock because of their high anthocyanin and starch contents and low lignin content [156, 157]. Under controlled laboratory conditions, induction of conversion to turion by abscisic acid treatment has been possible for *S. polyrrhiza* [156].

According to diverse goals and targets, optimal cultivation of duckweeds will be necessary from an economic and industrial point of view. Various culture methods of duckweeds using diverse types of bioreactors and conditions should be employed for the utilization as food, pharmaceutical, phytoremediation, and biofuel resources. Aquaponics that combine aquaculture and hydroponics could be a sustainable production system for plants [158]. Additionally, the cultivation of various plants employing the Internet of Things (IoT) and artificial intelligence (AI) technology enables the mass-production of good-quality plants by controlling environmental conditions, such as irrigation irradiation, atmospheric pressure, wind speed, temperature, and humidity [159, 160]. Aquaponics coupled with IoT and AI technology could be employed for duckweed cultivation in the future.

## Data Availability

Not applicable.

## Abbreviations

*L*.: *Lemna*
*S*.: *Spirodela*
*W*.: *Wolffia*
WHO: World Health Organization
LEX System: Lemna Expression System
IoT: Internet of Things
AI: Artificial intelligence
